# Homogenizing cellular tension by hepatocyte growth factor in expanding epithelial monolayer

**DOI:** 10.1038/srep45844

**Published:** 2017-04-04

**Authors:** Hwanseok Jang, Jacob Notbohm, Bomi Gweon, Youngbin Cho, Chan Young Park, Sun-Ho Kee, Jeffrey J. Fredberg, Jennifer H. Shin, Yongdoo Park

**Affiliations:** 1 Department of Biomedical Sciences, College of Medicine, Korea University, Seoul 02841, Korea; 2 Department of Environmental Health, Harvard T.H. Chan School of Public Health, Boston, MA 02115, USA; 3 Department of Engineering Physics, University of Wisconsin-Madison, Madison, WI 53706, USA; 4 Department of Biomedical Engineering, Hanyang University, Seoul 04763, Korea; 5 Department of Mechanical Engineering, Korea Advanced Institute of Science and Technology, Daejeon 34141, Korea; 6 Department of Microbiology, College of Medicine, Korea University, Seoul 02841, Korea

## Abstract

Hepatocyte growth factor (HGF) induces cell migration and scattering by mechanisms that are thought to tip a local balance of competing physical forces; cell-to-cell and cell-to-substrate forces. In this local process, HGF is known to attenuate local cadherin-dependent adhesion forces for cell-cell junction development and enhance local integrin-dependent contractile forces for pulling neighboring cells apart. Here we use an expanding island of confluent Madin-Darby canine kidney (MDCK) cells as a model system to quantify the collective cell migration. In the absence of HGF, cell trajectories are highly tortuous whereas in the presence of HGF, they become far less so, resembling free expansion of a gas. At the level of cell-to-cell junctions, HGF attenuates the linkage of stress fibers to cell-to-cell junctions with concomitant decrease in intercellular stress. At the level of cell-to-substrate junctions, HGF augments the linkage of stress fibers to cell-to-substrate junctions with no apparent effect on traction. Together, HGF induces both structural changes in the actin-bound junctional protein complex and physical forces spanning multicellular clusters, which further promotes the expansion of confluent cellular layer.

Within an epithelial layer, cellular migration is a collective process that is known to impact development, wound healing, remodeling, and metastasis^1^,^2^. Such cooperative movement within a multicellular unit is coordinated not only by biochemical signaling but also by physical interactions with underlying substrates and neighboring cells^3^. These physical communications include traction forces at cell-substrate adhesion sites and intercellular stresses at cell-to-cell adhesion sites^4^. The physical stresses are mainly generated by actomyosin machineries and are transmitted through cytoskeleton networks and adhesion molecules such as cadherins and integrins^5^. These physical stresses and the structural molecules must be dynamically orchestrated to maintain coordinated collective motions in cell clusters^6^,^7^.

To study collective migration in epithelial cells, hepatocyte growth factor (HGF), also referred to as scatter factor, can be used to reduce the collectiveness by influencing the proteins in the junctional complexes^6^,^8^,^9^. Recent studies showed that HGF disturbed the cell-cell junctions not by suppressing E-cadherin expressions^9^ but by strengthening the focal adhesions and associated integrin-dependent actomyosin activity, which acted in concert to pull apart the neighboring cells^10^. These events can lead ultimately to the epithelial-mesenchymal transition (EMT)^11^,^12^. Though the effect of HGF has been studied extensively *in vitro* focusing on clusters of a few cells^10^,^13^,^14^, or the boundaries of cell monolayer^15^,^16^, how HGF works within monolayer has not been studied in depth.

To fill this gap, we shift the focus to the multicellular dynamics that play out deep within the confluent cellular layer far from the layer margins. Using the methods described below, we studied Madin-Darby canine kidney (MDCK) cells that were grown to confluence within a large confined island (700 μm diameter containing approximately 1000 cells), and the dynamics that ensue when confinement is suddenly released and the layer becomes free to expand into unoccupied space. In that system with and without exposure to HGF, we have mapped in detail the cellular trajectories, the tractions that are exerted by the layer upon its substrate, and the stresses that are transmitted within the cell layer itself across cell-cell junctions. These biophysical quantities were complemented by mapping of the distribution of f-actin, E-cadherin, and vinculin. Together, resulting data suggest that cell scattering is dominated not so much by local events at the cell cell-junction, as previously believed, as much as by cooperative forces that are transmitted from cell to cell and span many cells.

## Results

### HGF converts cellular trajectories from meandering to persistent

**Figure 1:**
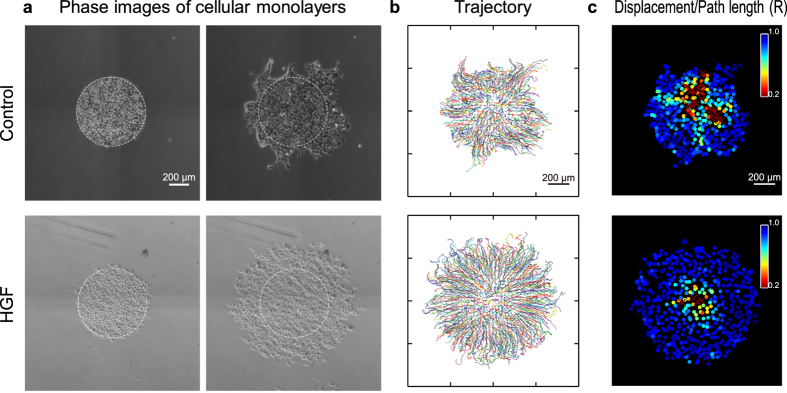
Expansion of MDCK cellular islands. (**a**) Phase contrast image of control and HGF-treated cellular islands at 1 hour and 12 hours after removing the mask. (**b**) Trajectory plots of individual cells in the islands for 12 hours after removing PDMS mask. (**c**) Ratio of displacement to path length for control and HGF-treated samples were examined for duration of 9 to 12 hours. Cool colors indicate straighter motion than warm colors. All data sets are representative of multiple experiments.

The cells in the monolayer should not scatter individually during expansion, but instead, should spread collectively throughout the experiment. To select the appropriate size of the pattern, we tested 300, 500, and 700 μm diameter islands. Within 5 hours, undesirable tearing of the monolayer was observed in the 300 and 500 μm diameter islands. The diameter of the island should be kept below 700 μm to ensure that it remains within the field of view during the expansion at a desired magnification. Based on these results, we only used a 700 μm diameter circular pattern containing approximately 1,000 cells. (Supplementary Fig. S2). When we removed the PDMS mask which was used to confine the cells into a circular region, the cells started to migrate radially toward free space (Fig. 1a left panel). Compared to the untreated controls, the HGF treated monolayers expanded much faster (Fig. 1, Supplementary movies 1, 2). After 12 hours, however, the boundaries of the untreated islands began to feature fingering instabilities with more tortuous peripheries, whereas the HGF treated islands did not (Fig. 1a). The effects of the HGF on the rate of expansion and the smoothness of the boundaries were dose-dependent (Supplementary Fig. S3). The higher increase in the areal expansion in the HGF treated sample was shown to correlate better with the increase in the projected area of individual cells rather than the number of cells in the island. At 10 ng/ml HGF there was a negligible effect on cellular proliferation (Supplementary Fig. S4a–d), leading us to conclude that the fast expansion of the HGF-treated island was not due to the increased proliferation but rather enhanced migration of cells comprising the layer.

Cellular trajectories were measured from displacement fields over 12 hours to identify differences in motion between the control and the HGF-treated island (Fig. 1b). Cells in the control island showed collective meandering behavior during the expansion whereas the cells in the HGF-treated island migrated centrifugally in relatively straight paths. As an index of the tortuosity of each trajectory, we measured the ratio of the displacement to the path length between the 9 and 12 hours (Fig. 1c). Colors in Fig. 1c represent path tortuosity, with warm colors indicating random migration and cool color indicating persistent migration. In the control samples, a high population of meandering cells appeared over a large area on the island. However, in the HGF treated sample, the cells at the very center exhibited more tortuous motion, presumably due to caging by neighboring cells, but otherwise the cells migrated in a straight path (Fig. 1c). Taken together, we found that during the island expansion HGF converted the migration of each cell from meandering to persistent.

### Within the cell layer HGF reduces tension build-up

**Figure 2:**
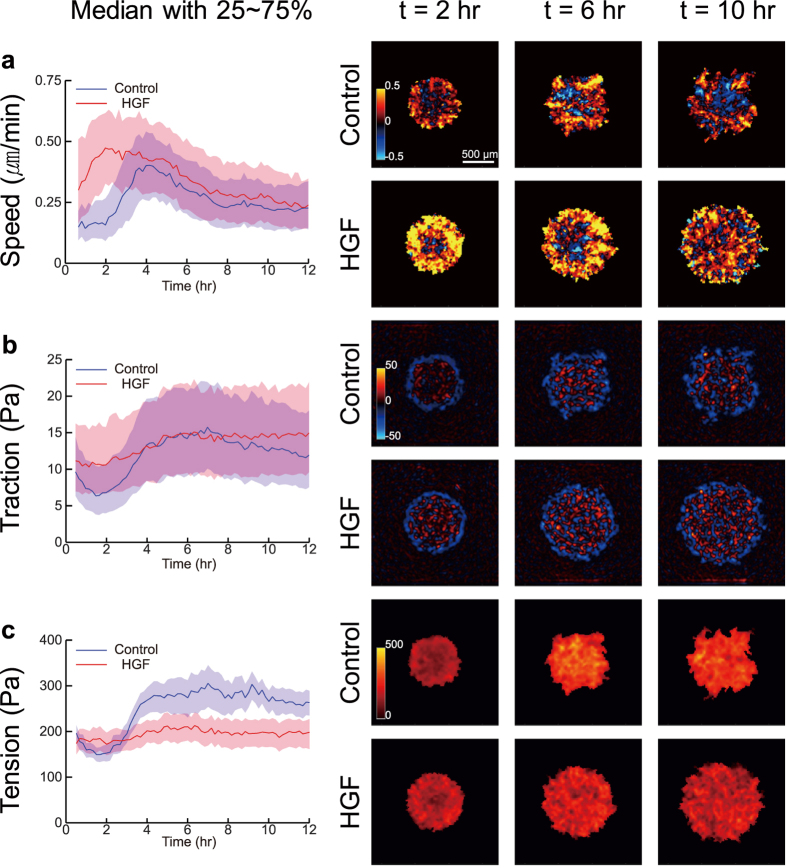
Distribution of speed, traction and tension in the cellular islands during the monolayer expansion. 25~75% interquartile ranges (shaded regions) and medians (solid lines) were computed for the whole cellular island and plotted over time (left panels). For spatial distribution, radial components of speed and traction and magnitudes of tension in the island measured at 2, 6, and 10 hours after removing PDMS mask were plotted in color coded maps (right panels). (**a** and **b**) As speed and traction are a 2D vector, from the center of the islands, radially inward components are shown in cool color, and radially outward components are shown in warm color. (**c**) Brighter indicate greater tension.

To investigate the effect of HGF on cellular mechanics, we assessed cellular velocities in relationship to traction stresses exerted by the cells upon the substrate and intercellular stresses exerted by cells on their neighbors (Fig. 2). We first plotted cellular speed, traction, and intercellular tension as a function of time (left panels), with the median of each expressed as a solid line and the 25~75% interquartile range expressed in a colored band. We also plotted color maps to visualize the spatial distribution of speed, traction, and tension. In the control islands, cellular speed slowly increased for 4 hours and then decreased for 8 hours to a plateau. In the HGF-treated islands, cellular speed increased more rapidly, reached a maximum at 2 hours, and by 12-hours slowly decreased to a level similar to that of the control sample (Fig. 2a). As shown in the color map, the control group maintained clusters of cooperative packs that moved either inward (cool color) or outward (warm color) within the island over time. On the contrary, HGF facilitated centrifugal migration (warm color) except for the central core of the island at the early time point (~2 hours). As time increased, cells primarily migrated centrifugally, but cellular clusters migrating inward began to scatter out from the center.

Because the cellular motions are regulated by physical forces between cell and its substrate and between cell and its neighboring cells, we measured traction and tension across the island using TFM and MSM. As shown in Fig. 2b, the traction in the HGF treated cells started off at a higher value and remained relatively constant over 12 hours. In the control sample, median traction values showed a transient hump at 1~3 hours, but the spread of traction values was similar to that of the HGF treated sample. A histogram showing the distribution of traction also indicated similar tendencies (Supplementary Fig. S5a). The overall spatial distribution of traction in the HGF sample showed more homogeneous distribution of pulling (blue) and pushing (red) forces over the entire time.

HGF had the most dramatic effect on intercellular tension (Fig. 2c). In the control sample, the initially weak intercellular tension strengthened over the course of 3 hours. Tension in the HGF-treated islands remained at a constant lower level over the entire period, which also was clearly evidenced in the frequency distribution shown in Supplementary Fig. S5b. Also, the color map of the control sample indicated strong accumulation of tension forces in clusters whereas the HGF samples showed no such characteristics.

### HGF decreases cellular cooperativity by modifying the intercellular tension

**Figure 3:**
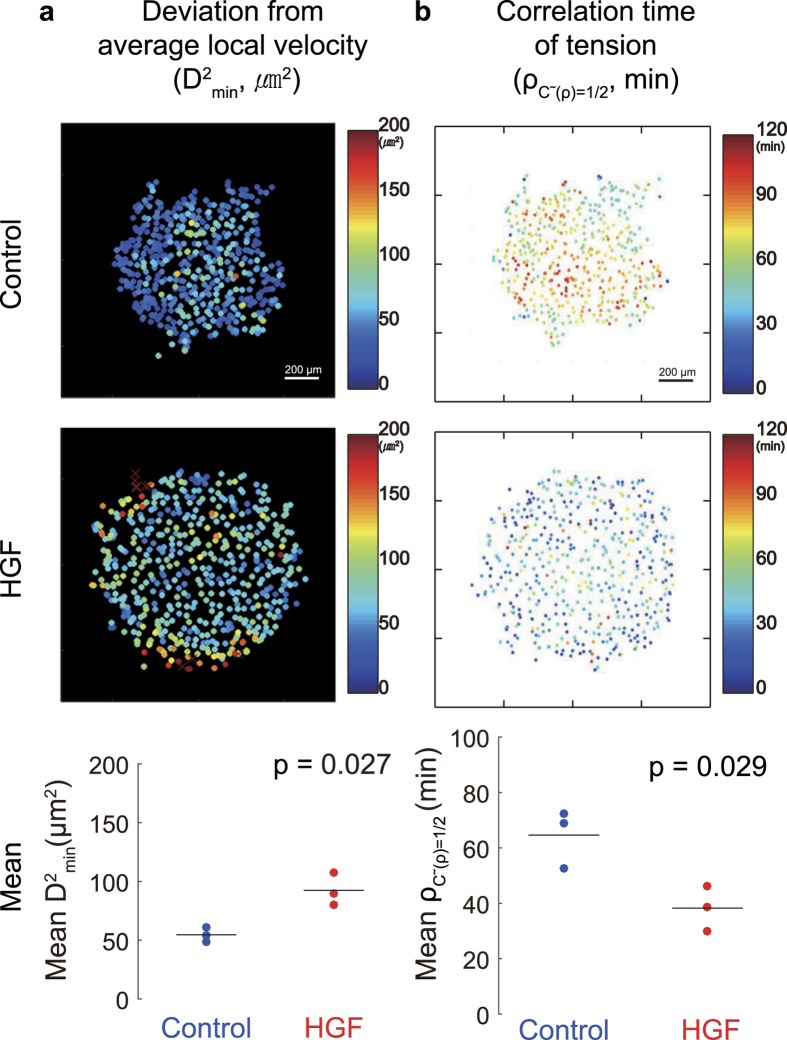
Cooperativity of motion and force in the cellular islands. Spatial distribution of cooperativity was examined for duration of 9 to 12 hours in color maps (upper panels) and averaged over all cells (lower panels, n = 3). (**a**) Deviations in motion between each cell and its 8 nearest neighbors are quantified with *D*^2^_*min*_. Dots in cool colors indicate cooperative cellular motions, whereas dots in warm color indicate individual, non-collective motions. (**b**) Correlation time of tension along cell trajectories, 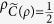. Dots in cool colors signify lower persistence—and therefore greater temporal fluctuations—in tension than dots in a warm color.

To quantify the cooperativity within the cellular island, we investigated how each cell’s motion deviated from that of its neighbors. To assess deviations in motion, we used the metric *D*^*2*^_*min*_, which represents how much each cell’s movement deviates from the average movement of its eight nearest neighbors^17^. Cells having a high value of D^2^_min_ move in a direction that is substantially different from their nearest neighbors. In the HGF-treated islands, the average value of *D*^2^_*min*_ was twice as large as for the control islands (Fig. 3a), which indicates that compared to control the cells in the HGF-treated islands had reduced cooperativity and migrated more independently than their neighbors. To test the hypothesis that the reduced cooperativity in motion was related to cellular contraction, we quantified fluctuations in contractile tension. For each cell we computed the tension over time and quantified fluctuations using a temporal autocorrelation. Results are presented as the scalar 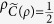, which is the time over which the correlation reaches a value of ½. A higher value of 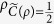 indicates that cellular tension remains at a relatively constant value for a longer period of time. The results show that the typical correlation time was shorter in the HGF-treated island than the control (Fig. 3b). These findings point to the notion that the treatment with HGF decreased cellular cooperativity in motion by modifying the intercellular tension.

### HGF modulates localization of actin-bound junctional proteins and attenuates the cooperativity of cellular motion and forces

**Figure 4:**
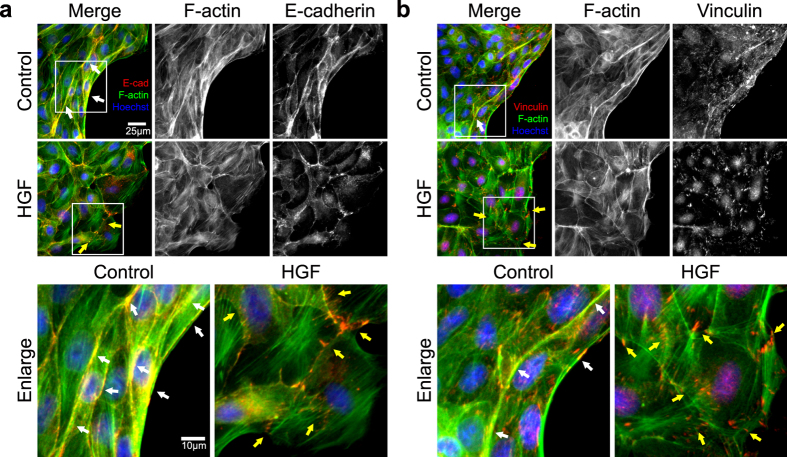
Immunofluorescence assays on tension-related machinery proteins in the cellular islands. To validate the interference of co-localization by HGF, an immunofluorescence assay was performed on E-cadherin and vinculin in the islands that were fixed 9 hours after removing PDMS mask. F-actin and cell nuclei were also fluorescently labeled. The white boxes in the upper panels are enlarged at lower panels. (**a**) E-cadherin was expressed as distinct and thick lines with co-localization with F-actin bundles in the control sample (white arrows) but was expressed as fuzzy and punctate zones with dissociated-F-actin in HGF sample (yellow arrows). (Red; E-cadherin, green; F-actin, and blue; Hoechst) (**b**) Vinculin was expressed as thin lines along with F-actin bundles (white arrows) and hazy dots linked at the ends of F-actin in the control sample but was expressed as dispersed and distinct dots along the individual cell boundaries in HGF samples (yellow arrows). (Red; vinculin, green; F-actin, and blue; Hoechst).

**Figure 5:**
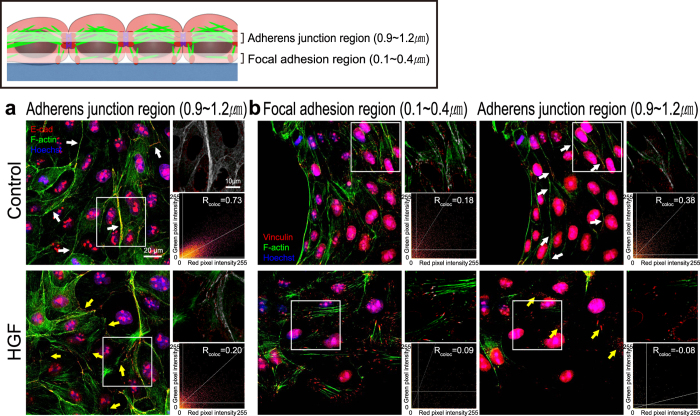
Confocal microscopy to investigate the localization of F-actin, E-cadherin and vinculin in the cellular islands. MDCK cell islands were imaged using confocal microscopy to examine the location of these proteins through the thickness. On the basis of nuclear location, the basal section (0.1~0.4 μm) was regarded as the region of focal adhesions (FAs), and the mid-apical section (0.9~1.2 μm) was regarded as the region of adherens junctions (AJs). As in Fig. 4, white arrows indicate co-localization of each protein and yellow arrows indicate dissociation. Representative regions used to examine co-localization (white boxes) are enlarged on the upper right panel (white; locations of co-localization) and presented in a scatter plot with a correlation coefficient line on the lower right panel. (**a**) E-cadherin was present only on AJ region because it was not expressed in FA region. (Red; E-cadherin, green; F-actin, and blue; Hoechst) (**b**) Localization of vinculin and F-actin showed differential expression depending on the z-position. F-actin and vinculin show lower expression in the AJ region in HGF sample compared to the control sample. (Red; vinculin, green; F-actin, and blue; Hoechst).

Based on the reduced magnitude and greater fluctuations in contractile tension caused by HGF, we hypothesized that HGF could directly alter cadherin-mediated adherens junctions (AJs) and actin filaments (AFs)^18^. The tension in the cellular island is also intimately associated with focal adhesion (FA) dynamics^19^. Therefore, in addition to cell-cell adhesion protein E-cadherin, we also investigated vinculin, a protein shared by both AJs and FA complexes^20^. We stained F-actin, E-cadherin, vinculin and Hoechst in the entire cell islands fixed at 3, 6, and 9 hours after expanding to visualize the global distribution of each protein in cell islands (Supplementary Fig. S6). The control islands showed well-developed continuous cortical actin cables over several cells along the periphery of the monolayer (Fig. 4a). On the contrary, cells along the periphery of the HGF-treated island exhibited distinct lamellipodia. E-cadherin in the control group is shown as solid lines along the cell-cell boundaries whereas that in the HGF-treated sample appeared less intact. In the case of vinculin, its accumulation on the cell-substrate interface is co-localized at the ends of stress fibers (Fig. 4b). In the HGF treated sample, we observed punctate FAs along the individual cell boundaries. In contrast, the control sample featured pronounced adhesion complexes predominantly on the leading edge with diffused small vinculin dots across the monolayer. Previous reports by le Duc *et al*. suggested that the spatial distribution of vinculin was regulated sensitively by the tensional state of the cells^6^. Because HGF apparently altered the inter- and intra-tension within the cellular monolayer, we decided to examine the exact location of vinculin using confocal microscopy. The typical height of the cells in our MDCK monolayer, in either the control or the HGF treated sample, was measured to be under 1.5 μm. We captured 15 images at 100 nm intervals and merged four images at the basal (0.1~0.4 μm) and in the mid-apical (0.9~1.2 μm) sections in order to create high quality representative projections of the FA region and the AJ region, respectively (Fig. 5). As shown in Fig. 5a, E-cadherin co-localized with the cortical actin at the cell-cell boundaries (white arrows) as continuous lines in the control cells, but E-cadherin appeared as dots along the actin in the HGF treated cells. As a result of co-localization analysis using ImageJ Colocalization plugin, white-colored regions in the enlarged images and scatter plots with PCC indicated that E-cadherin and F-actin were located at the same location in control sample whereas co-localization of two proteins was lower in in HGF-treated sample. In order to identify translocation of vinculin, we imaged both FA and AJ regions (Fig. 5b). As shown in Fig. 4b, vinculin was present in the FAs in both control and HGF-treated samples, but the expression was much stronger in the HGF-treated sample. Intriguingly, the AJ region of the control sample clearly indicated the localization of vinculin along the cell-cell junctions, co-localized with cortical actin (white arrows). HGF treatment apparently hindered the translocalization of vinculin to the AJ sites, explaining the presence of substantial basal accumulation of vinculin dots. As a result of the co-localization analysis for F-actin and vinculin, co-localized pixels (white) of the control sample image were mainly co-localized at the side regions of F-actins in the FA and linearly on the F-actins in the AJ. However, in the HGF-treated samples, the white-colored pixels were partially shown at the tips of F-actins in the FA and were seldom shown in the AJ. PCC values also clearly correlated with the extent of co-localization. Taken together, we suggest that HGF interfered with co-localization of the E-cadherin-vinculin-F-actin complex: HGF treatment induces an increase in intracellular stress fibers linked with FAs and a decrease in the multi-cell connected intercellular cortexes with AJs, leading to the disappearance of the cooperativity of cellular motion and forces in the cellular island.

## Discussion

Quantitative analysis of collective cell migration is of great significance in explaining various biological phenomena such as development, morphogenesis, and wound healing by analyzing the characteristics of multiple cells as a single entity. Many researchers studied the cellular monolayer by controlling conditions of cell clusters and their environments, and quantitatively measured the cellular behaviors aspect^15^,^21^,^22^,^23^,^24^. In this study, to quantify the epithelial expansion, we selected a circular island of an optimal size and the measured spatiotemporal change of cellular motions, forces and proteins in the environment with or without growth factor.

HGF plays an important role in collective cellular migration during invasion, morphogenesis and scattering, but how it affects the cooperative motions of cellular collectives has not been fully understood. Here we report that global heterogeneity in the motions and stresses within the expanding island are cooperative, but can be homogenized by HGF. HGF enhanced cellular migration speed while decreasing the correlated motions between neighboring cells to induce more persistent migration of individual cells. These results indicated that HGF-treated cells migrated more independently with minimal intercellular tugging interactions, exhibiting motions that are distinct from the typical ‘follow the leader’ type. In previous studies on the wound model, HGF treatment was shown to induce leader-like cells over the entire monolayer of MDCK cells^15^,^16^.

We demonstrated, further, that the HGF-induced suppression of cellular cooperativity was a functional consequence of reduced intercellular tension during the island expansion. While the tension within the cellular clusters in control sample accumulated within 3 hours to form the clusters of high tension, the HGF treatment interrupted the tension buildup across the entire island by compromising the spatiotemporal persistence of the tension. The median tension in the HGF treated island was maintained at a basal level over the entire period of experiments (12 hours). What is generally accepted is that the intercellular tension results from a two-dimensional imbalance between itself to the cell-to-substrate traction across the monolayer^25^. Therefore, one might expect to see a consistent pattern in tension and traction. However, unlike the clear difference in tension, the average cellular traction in the HGF sample was not different from that of control sample over time. Instead, the spatial distribution of traction showed distinct features between two samples. Thus, HGF-induced changes in the intercellular tension were transmitted as a consequence of altered local tractions values and inter-cellular cooperativity.

Furthermore, the dramatic reduction in the intercellular tension induced by HGF would be expected to correlate closely with functional status of the cell-cell junction. Previous reports showed the effective suppression of the cell-cell force transmission at the E-cadherin complex was effectively suppressed by HGF-treatment or Snail-overexpression^26^. This study demonstrated the HGF-induced inhibition of vinculin phosphorylation at Y822, suggesting possible cross-talk between the E-cadherin and vinculin. Our results also showed that the HGF treatment triggered the dissociation of E-cadherin as well as the decoupling of vinculin at the cell-cell junctions. More specifically, HGF weakens cell-to-cell connectivity via relocating vinculin from cell-to-cell junctions to cell-to-substrate junctions with attenuated intercellular tension, thereby reducing the cooperative pack.

**Figure 6:**
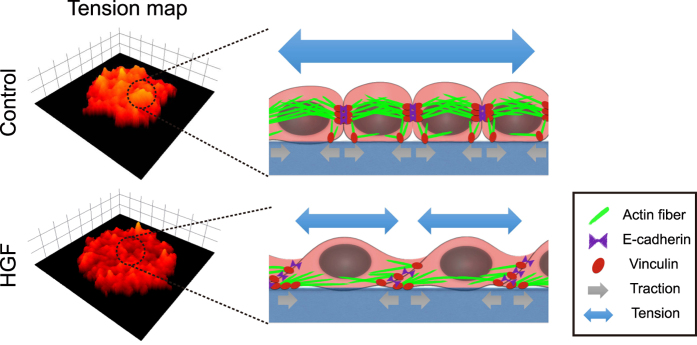
Tension buildup is modulated by HGF. HGF disrupts long-ranged actin stress fibers and induces dissociation of cell-cell adhesion complexes. The 3D-surface plots show the landscape of tension. Brighter and higher locations indicate greater tensions, which is supported by the connection between adhesion complex proteins and actin bundles.

In addition, HGF significantly altered actin organization. In the control sample, we observed cortical actin around individual cells along with stress fibers that spanned across multiple cells. In particular, the periphery of the expanding control islands featured thick and long actin cables alongside the several cells. In the HGF treated samples, on the other hand, the cortical actin appeared weaker with more pronounced yet shorter stress fibers connecting well-developed FAs. Because assembly of the AJ complex would require tension within the actin filaments and vice versa (36), the long-ranged stress fibers in the control sample are likely associated with the long-range tension-buildup through cell-cell adhesion complexes (Fig. 6). Under this model, disrupting either the junctional proteins or actin stress fibers would lead to attenuation of tension in the monolayer, as confirmed by our experiments (Fig. 2c).

Although a complete picture of the mechanism by which HGF changes the cooperative cell-cell interactions in the monolayer remains to be determined, our study has clearly shown that HGF acts on cellular machineries such as cadherin and vinculin to modulate the generation and distribution of force. This study confirms that the dynamic fluctuations and spatial heterogeneity in the cellular tension are determined by cooperative development of actin-associated adhesion complexes in the expanding cellular monolayer. The effects of HGF were not consistent with any local process, and instead reflected attenuation of cooperative processes that span many cells. The attenuation of collectiveness correlates with the loosening of the initially crowded cell pack. There are increasing recognition that this non-local cooperative process can be explained by the concept of jamming which have been proposed at the soft matter physics. Similarly to close-packed inert particulate matter, the increase or decrease in cell-cell adhesion would promote jamming or unjamming transition in the confluent living cellular layer^27^,^28^. In the context of jamming transition, we found that HGF interfered with the jamming of the cell collectives and promoted the expansion of a confluent layer by changing the cell-cell adhesive stresses and kinematic behaviors, consistently with the cellular “unjamming”.

## Materials and Methods

### Cell culture

Madin-Darby canine kidney (MDCK, strain II) cells were cultured in low glucose Dulbecco’s Modified Eagle’s medium (DMEM; Gibco) with 10% fetal bovine serum (FBS; Gibco) and 1% penicillin and streptomycin.

### Preparation of polyacrylamide gel substrates

Cells were cultured on masked polyacrylamide (PA) gel substrates (Young’s modulus = 6 kPa, thickness = 100 μm) that were prepared following the protocols in previous publications^22^,^29^. Briefly, the PA gel imbedding red fluorescent beads (diameter = 0.5 μm; FluoSpheres; Life Technologies) was polymerized in a silane-treated glass bottom dish (no. 1.5; *In vitro* Scientific) under a cover slip (diameter = 18 mm; VWR). The PA gel surface was functionalized with Sulfo-SANPAH (sulphosuccinimidyl-6-(4-azido-2-nitrophenylamino) hexanoate; 1 mg/ml in 50 mM HEPES buffer; Pierce) for immobilization of 10 μg/ml collagen type I (rat tail; Corning).

### Fabrication of PDMS masks

Cellular islands were patterned using thin polydimethylsiloxane (PDMS; Sylgard 184; Dow Corning) membranes with 700 μm-diameter holes. The SU-8 master mold (of 250 μm-thickness) for the PDMS mask was made-to-order at MicroFIT, Korea. PDMS prepolymer solution was poured on the mold and spun for uniformity. The PDMS was cured at 85 °C for 2 hours and then the PDMS membrane detached from the mold was trimmed with a circular punch of 16 mm-diameter. The resulting PDMS mask was autoclaved and coated with 2% Pluronic F-127 solution (diluted in PBS; Sigma-Aldrich) at 37 °C for 1 hour.

### Patterning cellular islands

Prepared PDMS mask covered on the gel substrate (Supplementary Fig. S1). The holes of PDMS mask were carefully filled with PBS so as to avoid air bubbles and a 200 μl drop of cell-suspension (density = 2 × 10^6^ cells/ml) was loaded in the holes. The sample was then incubated at 37 °C/5% CO_2_ for 1 hour. When the cells settled down on the substrate, the PDMS mask was carefully removed and the gel surface was washed twice with DMEM. Then, 3 ml normal DMEM or DMEM containing HGF (Hepatocyte Growth Factor; 10 ng/ml; Sigma-Aldrich) was added in the sample.

### Time-lapse microscopy

All experiments were performed on a DMI 6000B microscope (Leica) equipped with a climate-controlled chamber (37 °C and 5% CO_2_). Phase-contrast and fluorescence images were taken every 10 min for 12 hours and with a 5x objective lens. At every time point, 4 partially overlapping images were taken in a 2 × 2 grid and stitched together to get an image of a whole cellular island by using the Grid Stitching plugin of ImageJ software (National Institute of Health).

### Particle image velocimetry (PIV)

Displacements of the cells and the fluorescent beads were calculated using particle image velocimetry software written in MATLAB (Mathworks Inc.). To compute cellular velocity, the optical intensity of each phase-contrast image of every time-point was compared and cross-correlated to the image of next time-point. To measure traction, we first obtained bead displacements by cross-correlating the bead image of every time-point to the reference image, same position clearing the cells by trypsin.

### Fourier transform traction microscopy (FTTM)

Tractions of the cellular islands were measured using the unconstrained Fourier transform traction microscopy (FTTM) with corrections for substrates of finite thickness, which was previously described by Butler *et al*.^30^ and in other previous reports^29^,^31^.

### Monolayer stress microscopy

To calculate monolayer stress, we used the approach detailed by Tambe *et al*.^25^,^32^. Intercellular stresses within the cellular island were calculated from the traction by applying Newton’s first law of motion; the change in intercellular stress is balanced by the traction (*T*_*i*_) across the cellular island. The internal stress tensor, *σ*_*ij*_(*x, y*), is converted to the principal stresses, *σ*_*max*_ and *σ*_*min*_, by eigenvalue decomposition. From the principal stresses, the local average normal stress is defined as (*σ*_*max*_ + *σ*_*min*_)/2, and it represents the scalar tension within the cellular monolayer.

### Computation of deviation from average local velocity

Following the method of Falk and Langer^17^, we measured the extent to which cellular rearrangements are locally non-affine by computing *D*^2^_*min*_ for each cell over a period of 100 min using the displacement fields of eight-nearest neighbors.


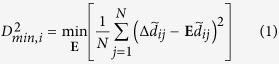



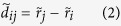



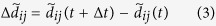


*i* represents a cell of interest, *j* as indexes of *N (N* = 8) nearest neighbors of cell *i*, and **E** as a 2 × 2 strain tensor representing a best-fit to the displacements of cells *i* and *j*.

### Computation of correlation time from correlation function of tension over time

From the tension, we computed an autocorrelation of a scalar variable magnitude over time for each cell trajectory.


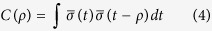



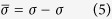


where, *ρ* is a specific time point and variable *t* is time from 0 to 720 min, which is the entire duration of the experiment. 〈*σ*〉 is a mean of tension *σ*. To normalize, *C(ρ)* is divided by *C(*0).


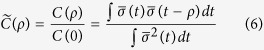


We defined the correlation time as 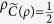, meaning the timespan over which the tension is relatively constant. To compute 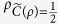, we found the point where 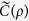 drops to a value of 1/2.

### Immunofluorescence assay

MDCK cell islands at 3, 6, 9 and 12 hours after releasing were pre-treated with 0.4% Triton X-100/0.4% paraformaldehyde in PBS for 3 min and fixed with 4% paraformaldehyde in PBS for 20 min. Cells were permeabilized with 0.4% Triton X-100 and blocked with 5% bovine serum albumin (BSA). The antibody for E-cadherin (24E10; 1:50; Cell Signaling) or Vinculin (hVIN-1; 1:400; Sigma-Aldrich) was treated to the cell islands. After washing the cells with PBST, Texas Red-conjugated secondary antibody, Alexa Fluor 488 phalloidin (1:200; Life Technologies) and Hoechst (33342; 1:3000; Life Technologies) were added for staining the cell islands. Cells were mounted in Elvanol reagent and covered with a cover glass (diameter = 12 mm; Marienfeld). Fluorescent images were taken with using a fluorescence microscope (Axioscope; Carl Zeiss) and a confocal microscope (LSM 510, Carl Zeiss). The confocal images were captured ~15 images at 100 nm interval per a position. In order to observe representative regions for focal adhesion and adherens junction, 4 images at the basal sections (0.1~0.4 μm) and in the mid-apical sections (0.9~1.2 μm) were respectively merged by projections of maximal intensity using ImageJ.

### Quantification of protein co-localization assay

To quantify co-localization of two proteins, intensity correlations between F-actin and E-cadherin or F-actin and vinculin were examined using ImageJ with trimmed-confocal images (47 μm × 47 μm field). The images were eliminated nucleus region to reduce noise by interference of fluorescence, and then red and green channels were used for analysis. Co-localization analysis was performed using ImageJ Colocalization plugins (Colocalization threshold and Coloc 2) as in previous studies^33^,^34^,^35^. Results of the co-localization analysis were shown by co-localized pixels of the examined images, scatter plots, and Pearson’s correlation coefficients (PCCs). Co-localized pixels of red and green channels were presented to white color in the merged images. The intensity of two color channels was plotted against each pixel in the scatter plots. Moreover, it was numerically quantified with PCC, which expresses the intensity correlation of co-localizing regions of the two color channels (+1 for perfect correlation, 0 for no correlation, and −1 for perfect anti-correlation).


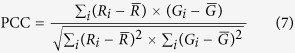


where, *R*_*i*_ and *G*_*i*_ represent the intensity values of the red and green channels, respectively, of pixel *i*. 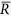 and 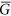 are the mean intensities of the red and green channels, respectively, of the entire image.

## Additional Information

**How to cite this article:** Jang, H. *et al*. Homogenizing cellular tension by hepatocyte growth factor in expanding epithelial monolayer. *Sci. Rep.* **7**, 45844; doi: 10.1038/srep45844 (2017).

**Publisher's note:** Springer Nature remains neutral with regard to jurisdictional claims in published maps and institutional affiliations.

## Supplementary Material

Supplementary Movie 1

Supplementary Movie 2

Supplementary Information
