# Supplementary material for: Homogenizing cellular tension by hepatocyte growth factor in expanding epithelial monolayer
Source: Sci Rep. 2017 Apr 4;7:45844. doi: 10.1038/srep45844 (PMC5379206; doi:10.1038/srep45844)
Supplement: Supplementary Information [file srep45844-s3.pdf]

Homogenizing cellular tension by hepatocyte growth factor in expanding epithelial monolayer

Hwanseok Jang,[1] Jacob Notbohm,[2,3] Bomi Gweon,[2,4] Youngbin Cho,[5] Chan Young Park,[2] Sun-Ho Kee,[6] Jeffrey J. Fredberg,[2] Jennifer H. Shin,[5,*] and Yongdoo Park[1,*]

[1] Department of Biomedical Sciences, College of Medicine, Korea University, Seoul 02841, Korea
[2] Department of Environmental Health, Harvard T.H. Chan School of Public Health, Boston, MA 02115, USA
[3] Department of Engineering Physics, University of Wisconsin-Madison, Madison, WI 53706, USA
[4] Department of Biomedical Engineering, Hanyang University, Seoul 04763, Korea
[5] Department of Mechanical Engineering, Korea Advanced Institute of Science and Technology, Daejeon 34141, Korea
[6] Department of Microbiology, College of Medicine, Korea University, Seoul 02841, Korea

* Authors for correspondence (ydpark@kumc.or.kr / j_shin@kaist.ac.kr)

Supplementary Information

Movie 1. Time-lapse of the controlled MDCK cellular island expanding from 1 hour to 12 hours after removing the mask.

Movie 2. Time-lapse of the HGF-treated MDCK cellular island expanding from 1 hour to 12 hours after removing the mask.

Figure S1. Schematics of patterning MDCK cellular islands. (a) The PDMS mask was placed on the PA gel for patterning MDCK cells. (b) A drop of cell suspension media (density = 2×10^6 cells/ml) covered the holes of the PDMS mask. (c) The PDMS mask was removed and cell remainders were washed out.

Figure S2. Size-dependence of cellular island expansion. Epithelial cell monolayer with the size of cell islands with 300, 500 and 700 μm diameter was cultured up to 10 hours and cellular scattering of different cell islands were observed.

Figure S3. Dose-dependence of HGF on cellular island expansion. The area of the cellular islands increased linearly with increasing HGF concentrations (0, 1, 10 and 100 ng/ml). Data are presented as mean ± SD from n=3 independent cell islands on each condition of HGF.

Figure S4. Kinematic analysis of the control (blue line with the blue square) and HGF-treated (red line with red circle) cellular islands over time. (a) Number of cells in each island. (b) Areas of the cellular islands. (c) The average number density of cells in the cellular islands. (d) The average area of each cell in the cellular islands. Data are presented as mean ± SD from n=3 independent cell islands at each time point.

Figure S5. Histograms of cellular traction and tension in the cellular islands. The frequency distributions were binned into four time periods (0~2 hours, 3~5 hours, 6~8 hours and 9~11 hours). A histogram is generated for each time period. The gray line shows a histogram for all times. (a) Histograms of traction applied by control and HGF-treated islands showed similar tractions at these time points. (b) A transition of the frequency distribution of tension occurred at an early time point in the control island, but not in the HGF-treated island. On average, tension was lower for the HGF-treated islands than the control islands.

Figure S6. Immunofluorescence assays on tension-related machinery proteins in the entire cellular islands. An immunofluorescence assay was performed on E-cadherin and vinculin in the islands that were fixed 3, 6, and 9 hours after removing PDMS mask. F-actin and cell nuclei were also fluorescently labeled. (Left; red; E-cadherin, green; F-actin, and blue; Hoechst / Right; red; vinculin, green; F-actin, and blue; Hoechst)

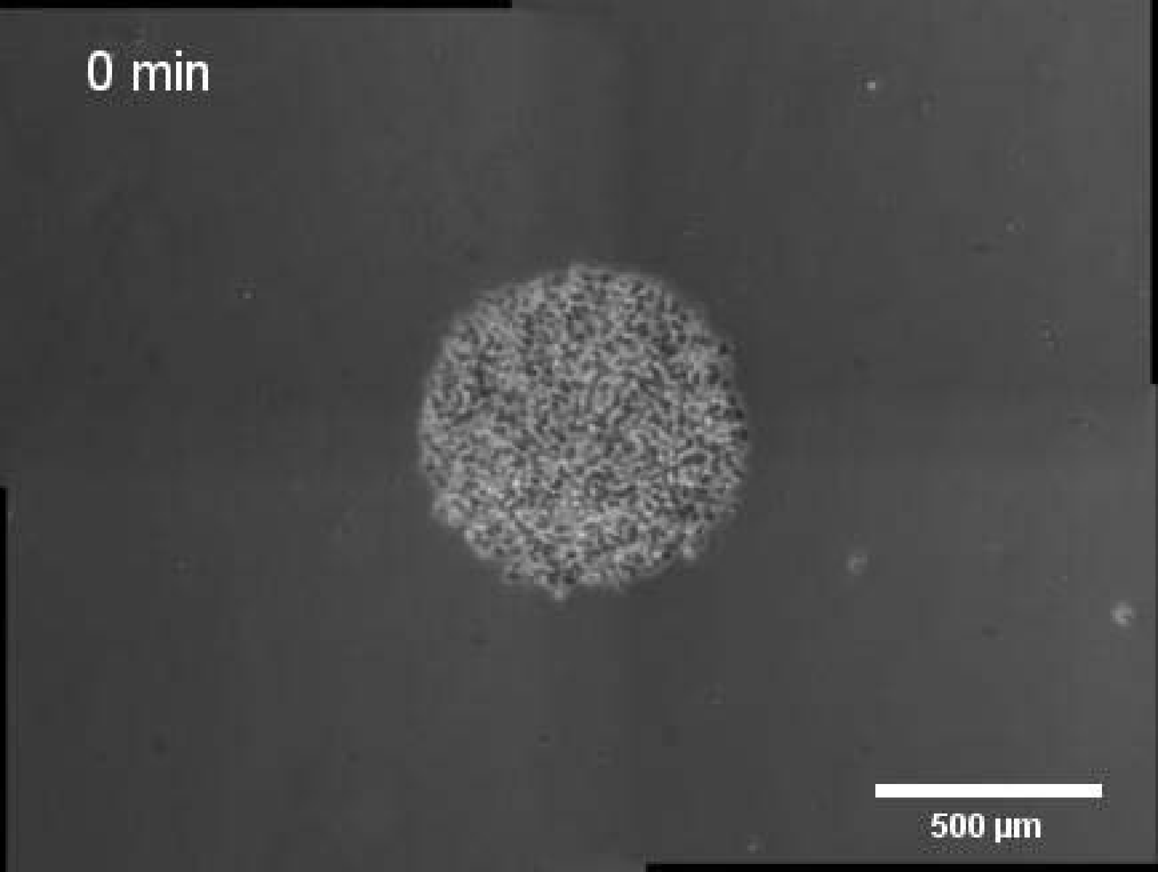
0 min
500 µm

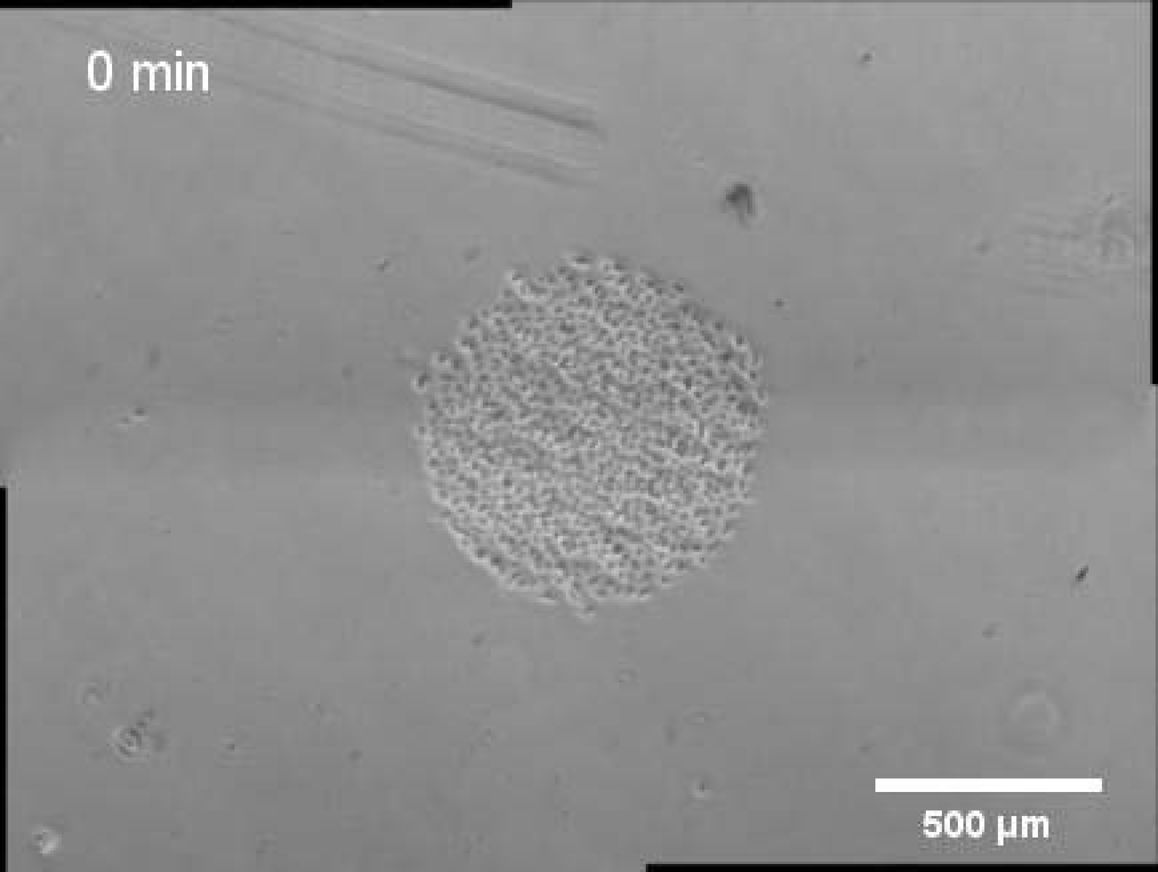
0 min
500 µm

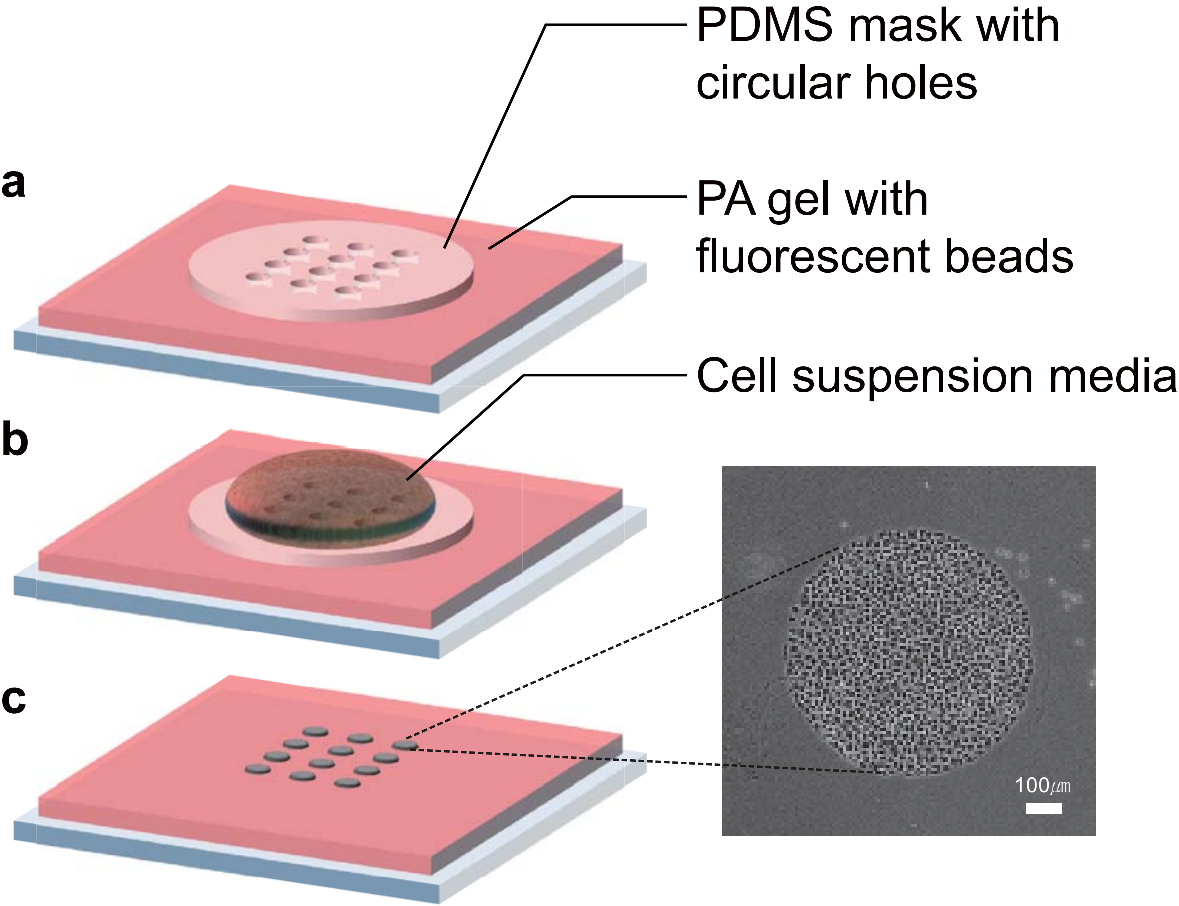
PDMS mask with circular holes
PA gel with fluorescent beads
Cell suspension media
a
b
c
100µm

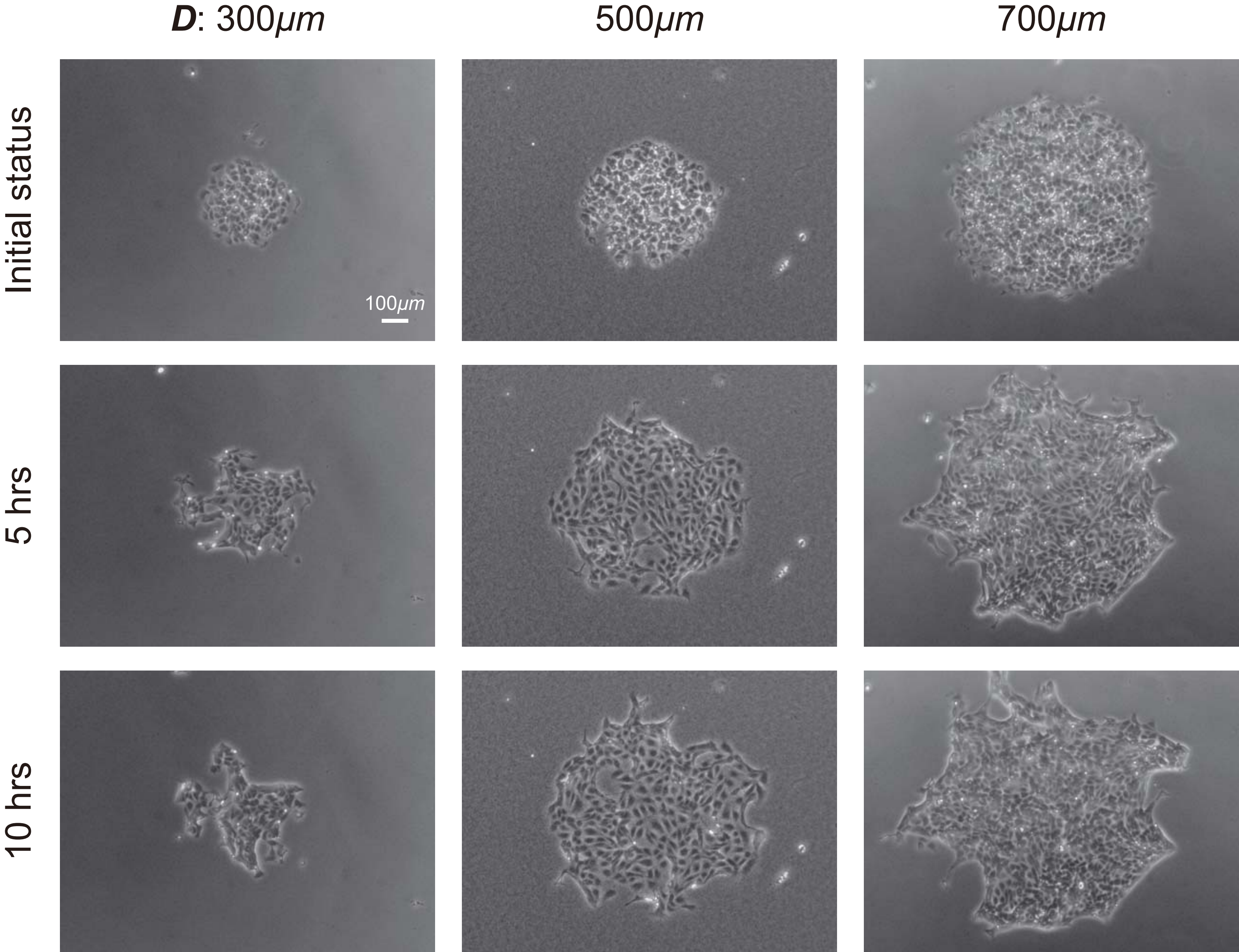

D: 300μm
500μm
700μm
Initial status
5 hrs
10 hrs
100μm

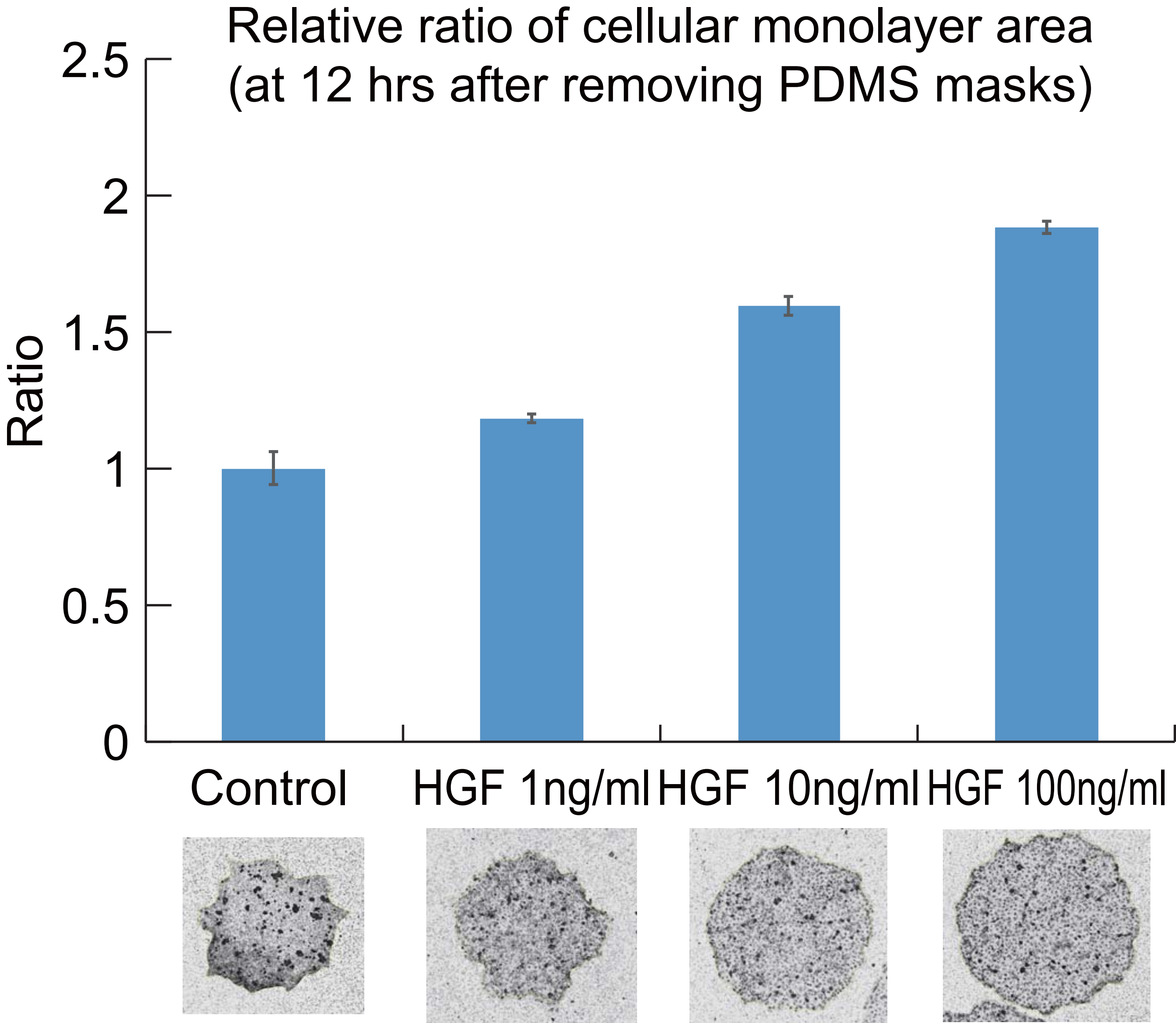
Relative ratio of cellular monolayer area
(at 12 hrs after removing PDMS masks)
Ratio
2.5
2
1.5
1
0.5
0
Control
HGF 1ng/ml
HGF 10ng/ml
HGF 100ng/ml

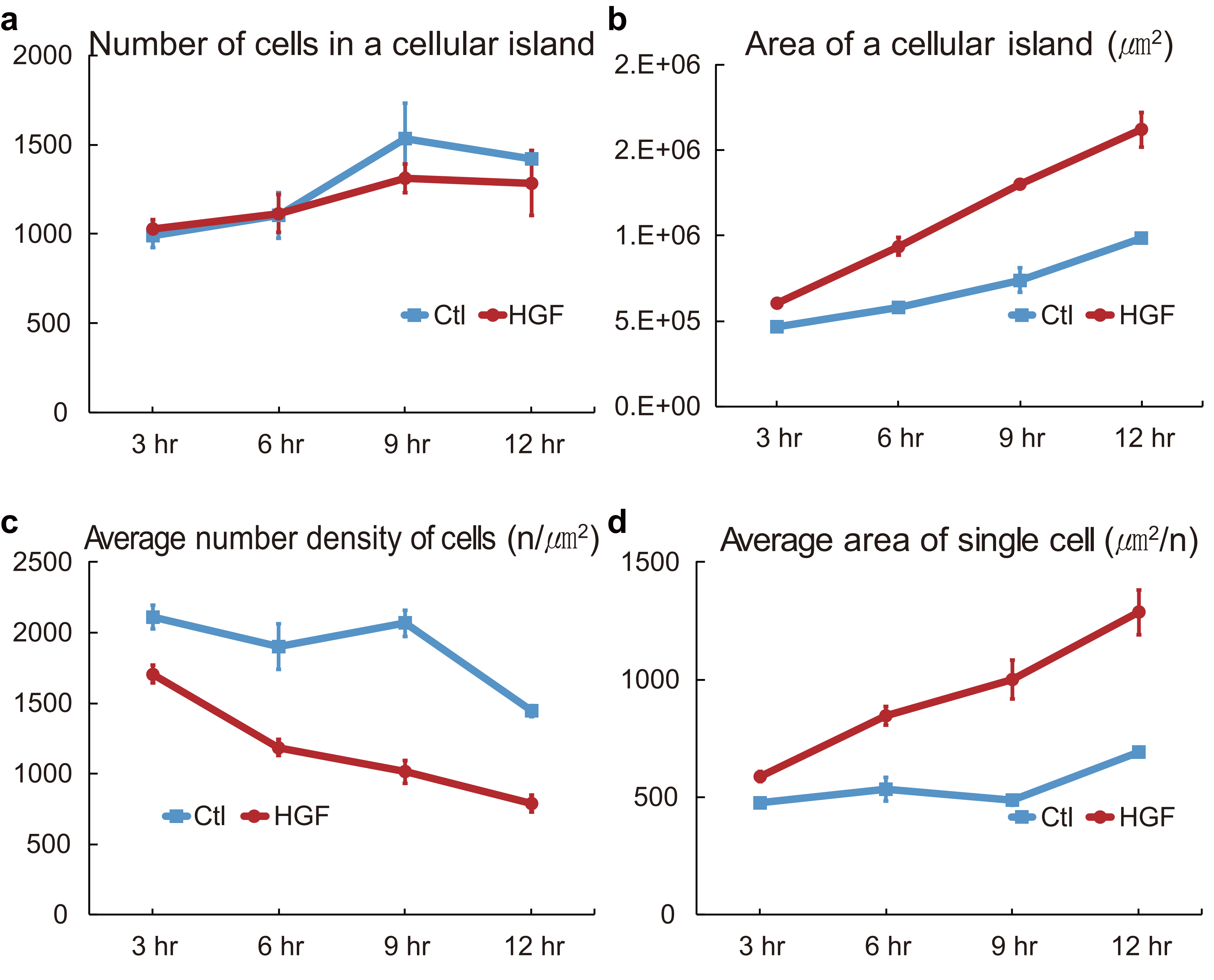

a
Number of cells in a cellular island
2000
1500
1000
500
0
3 hr
6 hr
9 hr
12 hr
Ctl
HGF
b
Area of a cellular island (㎛²)
2.E+06
2.E+06
1.E+06
5.E+05
0.E+00
3 hr
6 hr
9 hr
12 hr
Ctl
HGF
c
Average number density of cells (n/㎛²)
2500
2000
1500
1000
500
0
3 hr
6 hr
9 hr
12 hr
Ctl
HGF
d
Average area of single cell (㎛²/n)
1500
1000
500
0
3 hr
6 hr
9 hr
12 hr
Ctl
HGF

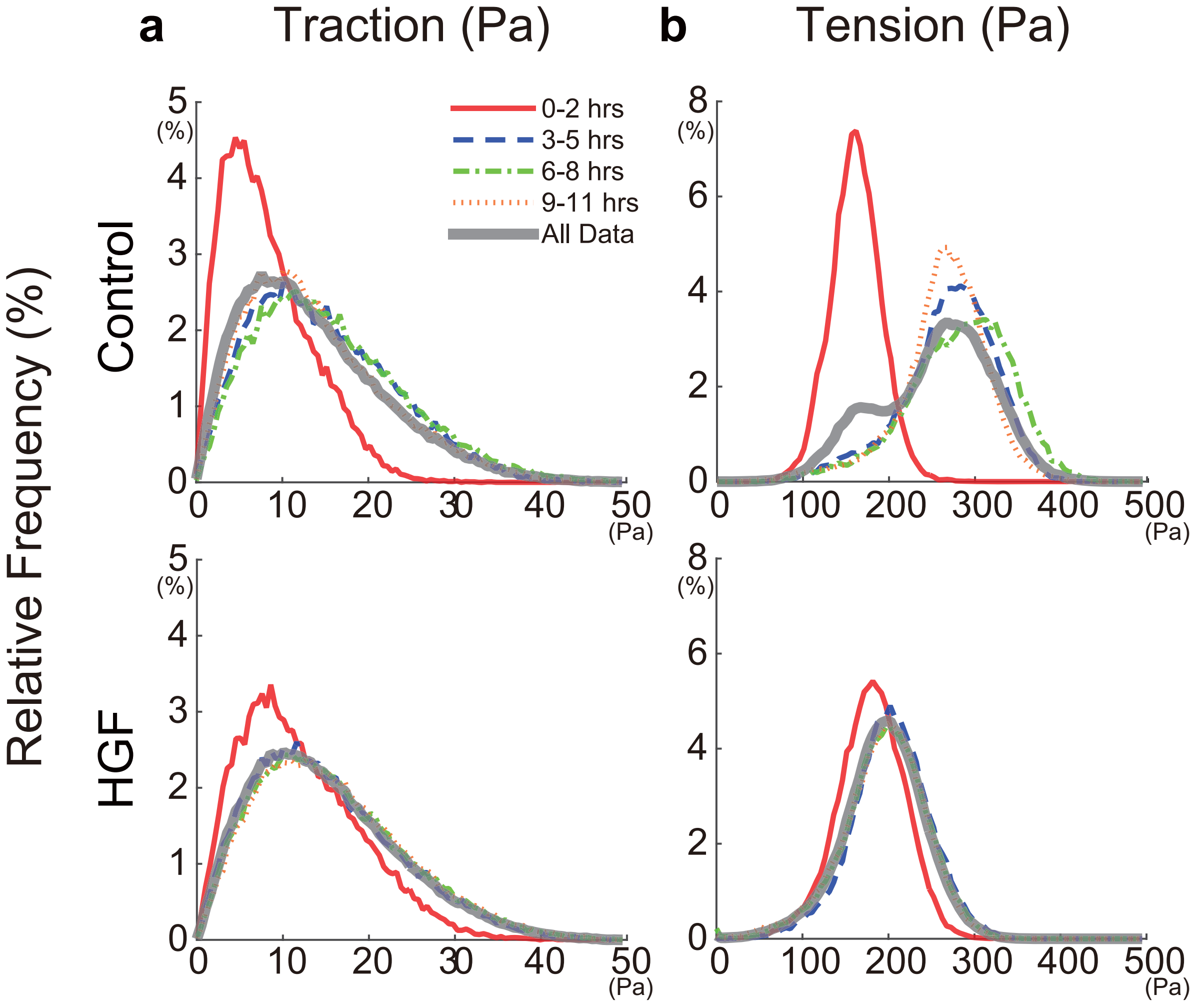
a
Traction (Pa)
b
Tension (Pa)
Relative Frequency (%)
Control
HGF
0-2 hrs
3-5 hrs
6-8 hrs
9-11 hrs
All Data
(%)
(Pa)
0
1
2
3
4
5
6
8
10
20
30
40
50
100
200
300
400
500

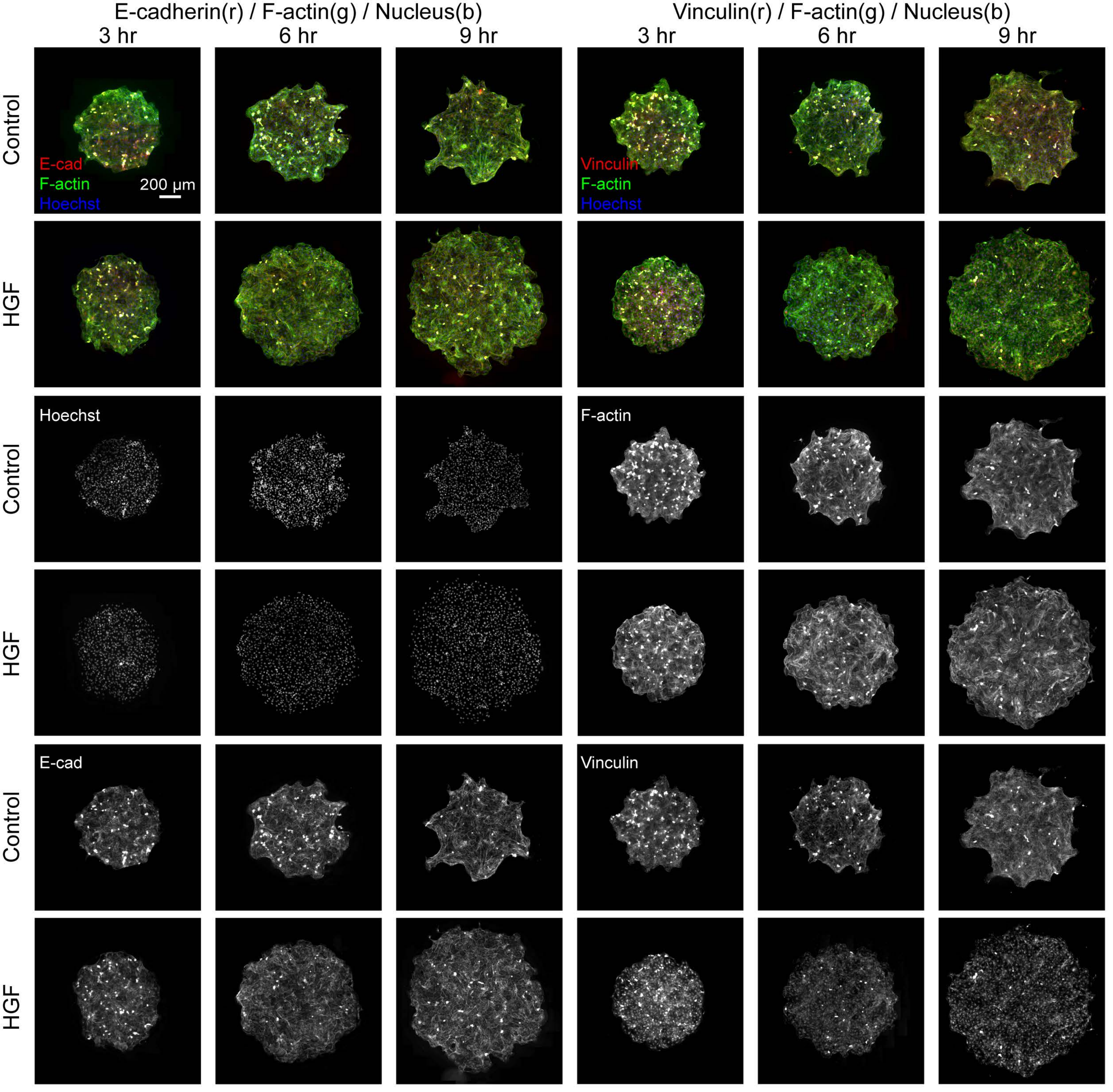

E-cadherin(r) / F-actin(g) / Nucleus(b)
Vinculin(r) / F-actin(g) / Nucleus(b)
3 hr
6 hr
9 hr
3 hr
6 hr
9 hr
Control
HGF
Control
HGF
Control
HGF
E-cad
F-actin
Hoechst
200 μm
Vinculin
F-actin
Hoechst
Hoechst
F-actin
E-cad
Vinculin
